# Genetic Admixture in the Population of Wild Apple (*Malus sieversii*) from the Tien Shan Mountains, Kazakhstan

**DOI:** 10.3390/genes12010104

**Published:** 2021-01-15

**Authors:** Young-Ho Ha, Seung-Hwan Oh, Soo-Rang Lee

**Affiliations:** 1 Division of Forest Diversity, Korea National Arboretum, Pocheon 11186, Korea; yh0990@korea.kr (Y.-H.H.); oshwan@korea.kr (S.-H.O.); 2 Department of Life Sciences, Gachon University, Seongnam 13120, Korea; 3 Department of Biology Education, College of Natural Sciences, 309 Pilmun-Daero, Dong-Gu, Gwangju 61452, Korea

**Keywords:** *Malus sieversii*, wild apple, hybridization, genetic swamping, genetic diversity, SSR

## Abstract

There is growing attention given to gene flow between crops and the wild relatives as global landscapes have been rapidly converted into agricultural farm fields over the past century. Crop-to-wild introgression may advance the extinction risks of rare plants through demographic swamping and/or genetic swamping. *Malus sieversii*, the progenitor of the apple, is exclusively distributed along the Tien Shan mountains. Habitat fragmentation and hybridization between *M. sieversii* and the cultivated apples have been proposed to be the causal mechanism of the accelerated extinction risk. We examined the genetic diversity pattern of eleven wild and domesticated apple populations and assessed the gene flow between *M. sieversii* and the cultivated apples in Kazakhstan using thirteen nuclear microsatellite loci. On average, apple populations harbored fairly high within-population diversity, whereas population divergences were very low suggesting likely influence of human-mediated dispersal. Assignment results showed a split pattern between the cultivated and wild apples and frequent admixture among the apple populations. Coupled with the inflated contemporary migration rates, the admixture pattern might be the signature of increased human intervention within the recent past. Our study highlighted the prevalent crop to wild gene flow of apples occurring in Kazakhstan, proposing an accelerated risk of genetic swamping.

## 1. Introduction

Inter-specific gene flow between rare species and the allied taxa that are commonly distributed may result in the increased extinction risk at least locally for the rare species [1,2,3,4]. Hybridization may lead to increased extinction risk through demographic swamping, i.e., reduced population growth rate, driven by outbreeding depression and/or genetic swamping where the parental lineages are replaced by the hybrids [2,5]. Recently, gene flow among related taxa have become more frequent due to habitat destruction and translocation by anthropogenic influences [6]. For plants, crop-to-wild gene flow is of special concern as it might jeopardize wild population’s integrity and intensify the extinction risk of the wild species [5,7,8]. Hybridization observed in *Prunus fruticosa*, a rare shrub distributed in Eurasia, with the widely cultivated cherries, *P. avium* and *P. cerasus* is one of the compelling examples showing likely risk of genetic swamping [9]. A recent review comprising over 350 hybridization-related studies also proposed that genetic swamping resulting from interspecific hybridization causes local extinction more frequently than what has been expected [5]. With the advent of many advanced molecular tools, examining the rate of hybridization in rare plants is feasible. The extent of hybridization estimated from molecular methods might offer valuable insights for conservation practices.

The genus *Malus* Mill. (Rosaceae) comprises about 25 to 47 species including one of the most important fruit crops, *M. domestica* [10]. The species delimitations and phylogenetic relationships among the taxa in the genus are complex in part due to the hybridization events that are prevalent throughout the genus *Malus* [10,11]. Hybridization between the cultivated apples (*M. domestica* Borkh.) and the two main contributors, *M. sieversii* (Ledeb.) M. Roem. and *M. sylvestris* (L.) Mill. is a well-recognized example of such taxonomic challenges [12,13,14,15,16]. Coupled with the shared morphological characters, the amount of genetic evidence also indicated that the cultivated apple is the descendant of the wild *M. sieversii* [15,16,17,18,19]. It is proposed that the wild progenitor, *M. sieversii* has primarily contributed to the genetic diversity of the apples along with a series of hybridizations and introgressions by secondary contributors (*M. sylvestris*, *M. baccata* (L.) Borkh. and *M. orientalis* Uglitz.) [8,16,18,20,21].

*M. sieversii* (Rosaceae, 2n = 34) is an insect pollinated long-lived tree that can grow up to 10 m and flower in May [20,22]. The species is mainly allogamous with high self-incompatibility [20,23]. *M. sieversii* can also vegetatively regenerate by stem shoots, adventitious root formation particularly under unfavorable conditions where the plants are physically damaged and/or competing on resources with neighboring plants [20]. The plant bears large fruits, comparable to domesticated apples (>60 mm) with diverse shapes and colors [15]. *M. sieversii* is exclusively native to the Tien Shan mountain range residing mostly mid elevation (900–1600 m) [15,20]. Within the last 30 years, over half of the natural habitats in Kazakhstan have rapidly declined due to farming, grazing and wood harvesting, posing great concern for local extinction [20,24]. Moreover, genetic swamping possibly caused by hybridization between *M. sieversii* and the cultivated apple may further aggravate the extinction risk. As apple farming expands in the regions, the natural habitats likely adjoin the local orchards shaping secondary contact zones. *M. sieversii* is currently listed as vulnerable on the International Union for Conservation of Nature and Natural Resources (IUCN) red list. Despite the great importance of conserving the wild apple populations, only a handful of studies examined the extent and impact of hybridization in the wild *M. sieversii* populations by domesticated apples [8,16,24,25]. A recent population genetics study of 16 microsatellites revealed that most of the wild *M. sieversii* populations showed a signature of hybridization with the apple cultivars [24]. Cornille et al. [8] also found a moderate proportion (~15%) of the domesticated apple’s introgression to *M. sieversii*. The results suggest the likely influence of hybridization and introgression of cultivated apples on wild *M. sieversii* populations.

In the present study, we aimed to determine the potential risk of genetic swamping resulting from gene flow between the cultivated apples and the wild progenitor (*M. sieversii*). Although caution must be taken [26], genetic data provide information that can infer hybridization as it leaves traceable marks in genomes [27]. We employed 13 microsatellite markers (SSRs) to assess the gene flow among natural *M. sieversii* populations and apple cultivars. The genetic diversity pattern of *M. sieversii* and how the wild populations spatially structured over heterogeneous landscapes were also investigated. Given the recent disturbances of natural habitats and the surge of agricultural activities, we hypothesized that there would be a high level of hybridization between the wild *M. sieversii* and cultivated apples, leading to a risk of genetic swamping. Genetic diversity is expected to be well maintained due to the potential hybrid events. Genetic studies often found an escalated infra-specific genetic diversity pattern in *M. sieversii*, possibly in association with closely related, *M. kirghisorum* and *M. niedzwetzkyana* [20,24,28,29]. Accordingly, we included *Malus niedzwetzkyana* Diek, an endangered congener that is closely related, in our analysis to assess the influence of the related taxa (*M. sieversii*). 

## 2. Materials and Methods 

### 2.1. Sample Collection and DNA Isolation

**Figure 1:**
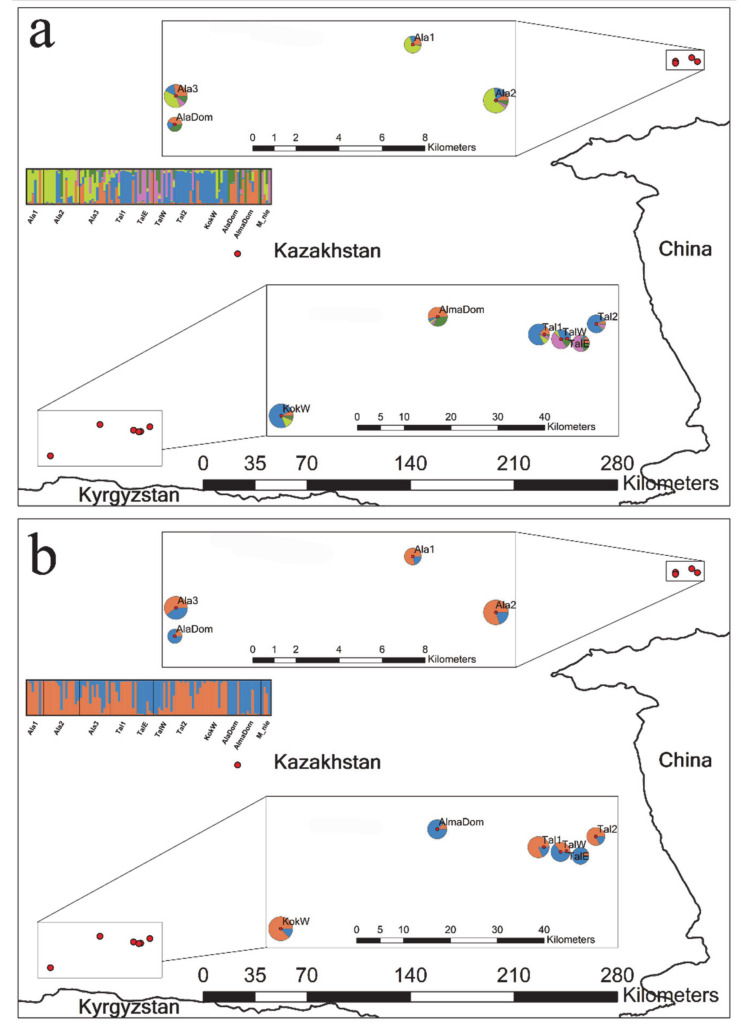
Results summary of Bayesian model-based clustering analysis based on 13 nuclear SSRs for eleven populations of the three *Malus* taxa. The bar plots show the group assignments of 102 individual genotypes for (**a**) K = 2 (the optimal number of clusters) and (**b**) K = 5 (the number of clusters with the second most likelihood; Figure S1). The vertical black lines separate populations. Pie charts on the map illustrate the frequency of each cluster for each population. See Table 1 for population abbreviations, sample locations and sample sizes.

We collected young leaves from 124 individuals of wild and domesticated apples (84 *M. sieversii*, 36 *M. domestica* and 4 *M. niedzwetzkyana*) across two consecutive springs of 2018 and 2019 from Kazakhstan (Table 1; Figure 1). The collected leaf tissues were then preserved at room temperature in plastic bags with silica-gel desiccants until further use. We randomly selected the sampling populations along the Tien Shan mountains in Kazakhstan. To avoid sampling from the populations that are too close, a minimum of 10-km distance was applied among the sampling populations. To carefully choose sampling populations for the wild apples, we used the documented records [24,30] and information provided by the local collaborator affiliated by Institute of Forestry and Agroforestry in Kazakhstan. Given the high rate of clonal propagation observed in the wild and the cultivated apples [20], we specified a minimum of 50-m distance among all the sampling individuals within each population. In the case of *M. niedzwetzkyana*, only four individuals were collected as the species inhabits a highly restricted area with a small number of individuals. We prepared at least two voucher specimens for each population and deposited those in the Korea National Arboretum herbarium, KHB (1602521: 1602666). All required permits were prepared and obtained by the local collaborator prior to the field sampling. 

Genomic DNA was extracted following the manufacturer’s protocol of the DNeasy Plant Mini Kit (Qiagen Inc., Valencia, CA, USA). The quantity and quality of extracted DNAs were evaluated in a NanoDrop ND1000 (Thermo Fisher Scientific, Waltham, MA, USA; quality cutoff, OD 260/280 ratio between 1.7–1.9) and visualized in 1% agarose-gel electrophoresis.

### 2.2. Nuclear and Chloroplast Microsatellite Genotyping

We amplified 17 SSR loci using the primer pairs described in three previous studies [31,32,33]. The seventeen SSR loci were carefully chosen from each of 17 chromosomes to ensure independence among all loci. PCR reactions were performed using commercially available AccuPower^®^ PCR premix (BIONEER, Daejeon, Korea) according to the manufacturer’s protocol. For multiplexing, we fluorescently labeled (FAM, HEX) the PCR products on the 5′-end of forward primers for every locus. The detailed amplification conditions and the information on the 17 SSR loci are provided in the supplementary information (Tables S2 and S3). The amplified fragments were separated out on an ABI 3730XL automated sequencer (ThermoFisher Scientific, MA, USA). We used the automated allele scoring on Microsatellites v. 1.4.6 implemented in Geneious R10 v. 10.2.6 (https://www.geneious.com) for the microsatellite profiling. The scoring results were then manually checked and corrected before finalizing the microsatellite profiles. We estimated the frequency of null alleles using INEST v. 2.2 [34]. 

### 2.3. Data Analysis

To avoid clone sampling, we removed the redundant genets from the analyses. For the clonality test, we used GenoDive v. 2.0b23 [35], whereby the frequency distribution of pairwise distances among all samples in a data set is utilized to set up an appropriate threshold defining clones. We used the distance between the first and the second peak as a threshold to assign the genotypes to clones. We excluded the 22 domesticated apple clones from the further analyses. With the finalized 102 samples, we estimated the genetic diversity parameters, He and Na, using Arlequin v. 3.5 and GENALEX v. 6.502 [36,37]. Due to the variation in the population sizes, we standardized the number of alleles to 8 loci, the minimum number of the loci in our data set, using rarefaction curves in HP-RARE [38]. Markers that significantly departure from the Hardy–Weinberg Equilibrium (HWE) may violate assumptions employed in coalescent based approaches such as Migrate-n and STRUCTURE. Thus, we tested for significant departures from HWE on 17 microsatellite loci within each population using Fisher’s exact test [39] in Arlequin. Bonferroni corrections for multiple comparisons (adjusted *p* values) were applied. As the 17 microsatellite markers were isolated from different chromosomes, we assumed that each marker is not in linkage. We filtered out four markers from all downstream analyses due to significant deviation from HWE (*p* < 0.01). Pairwise F_ST_ between all population pairs was calculated in Arlequin using 1000 permutations to ensure statistical robustness. We conducted a Mantel Test to examine Isolation by Distance using the pairwise genetic distances (Slatkin’s linearized F_ST_ = F_ST_/(1 − F_ST_)) and log-transformed Euclidean distances for all population pairs in GENALEX [37,40]. The statistical significance of the correlation coefficient (r) was tested by 1000 random permutations. 

We employed a Bayesian model-based clustering approach to examine population structure using the correlated allele frequencies model with admixture [41] implemented in STRUCTURE v. 2.3.4 [42]. Ten independent STRUCTURE runs were repeated for each K from 1 to 10 with the 1,000,000 MCMC (Markov chain Monte Carlo) iterations following 100,000 steps as burn-in. The optimal number of clusters (K) were inferred based on ΔK followed by Evanno et al. [43]’s method using STRUCTURE HARVESTER v. 0.6.94 [44]. We finalized the ancestry coefficients of 10 STRUCTURE repeats in CLUMPP v. 1.1.2 with the greedy option [45]. The final results were then visualized in DISTRUCT v. 1.1 [46]. The clustering pattern also was investigated through Principal Coordinate Analysis (PCoA). We performed PCoA using the pairwise Nei’s genetic distances estimated for all 102 individuals in GENALEX. The hierarchical molecular variance, AMOVA, was partitioned to within and between clusters identified by both STRUCTURE and PCoA in Arlequin. We ensured the statistical robustness by comparing the estimated values against values calculated from 1000 bootstraps. 

Because we aimed to examine the level of crop to wild gene flow, both historical and contemporary migration rates among the wild apple populations and the cultivated apples were estimated as a proxy for the gene flow. To avoid the computational challenges and parameter calculation biases, we redefined groups based on the genetic clusters inferred from the STRUCTURE (Figure 1; see Table 1 for the four groups). Historical migration rates among the four rearranged groups were calculated in a coalescent-based approach, MIGRATE-N 3.6.11 [47]. We assumed asymmetric migrations (M) among populations and computed M as m/μ, where m indicates an immigration rate per a generation and μ refers to a mutation rate [48]. An effective population size θ (4Neμ, where Ne = effective population size; μ = mutation rate) was also estimated in MIGRATE-N [48]. With the Bayesian approach, we ran three independent replicates using the Brownian motion mutation model. For the prior distribution of the parameters θ and M, a uniform model was used with minimum, mean, maximum, delta and bin values of 0, 50, 100, 10 and 1500, respectively. The run used 10,000 long-samples with an increment of 100 (1,000,000 iterations) after a burn-in of 100,000. Four heating chains were applied with swaps. The contemporary migration rates among the four groups were calculated in BAYESASS 3.0.1 [49]. We ran BAYESASS starting with a 1,000,000 burn-in followed by 10,000,000 MCMC iterations sampling every 2000. The default setting was used for the mixing parameters. To directly compare historical (M) and contemporary migration rates (m), M estimated from MIGRATE-N was adjusted by mutation rate, 10^−4^/allele/generation [50]. The mutation rate was employed based on the observed mutation rates for microsatellite markers in Arabidopsis thaliana [51]. We evaluated the statistical significance using a permutation test with 1000 replicates for the differences between the two migration rates in R 3.5.2 [52]. 

Historical and recent bottlenecks were examined using the Garza–Williamson index (G-W index; M-ratio) implemented in Arlequin and BOTTLENECK v. 1.2.02 [53,54], respectively [55]. The M-ratio test computes the ratio of the number of alleles to the allele size range as bottlenecks supposedly show reduction in the allele numbers more rapidly than the allele size ranges [54]. BOTTLENECK detects relatively recent declines, i.e., within the last few generations, in population sizes looking for the significant excess or deficit of heterozygosity that cannot be seen at equilibrium state [56]. We used the infinite allele model (IAM) and stepwise mutation model (SMM) for the BOTTLENECK run with Sign and Wilcoxon’s sign rank tests for statistical significance. Due to the limited number of samples collected for M_nie, the population was excluded from the bottleneck analyses. 

## 3. Results

There was no scoring error found in the 17 nuclear microsatellite profiles. Of the seventeen SSRs, null alleles were observed in five loci (loci 3, 4, 6, 11, 17) with low to moderate frequencies (null allele frequencies <0.3). However, null alleles were only present in one population for two (loci 4 and 6) of the five loci, thus those loci remained in the downstream analyses. Among the wild apple samples (*M. sieversii* and *M. niedzwetzkyana*), identical clones were not observed. However, over half of the domesticated apples turned out to be clones in both the two apple populations (5 of 10 from AlaDom; 17 of 26 from AlmaDom; see Table 1 for the population abbreviations). Thus, we screened the 22 clones out, finalizing 102 genotypes for further analyses. Four loci (3, 6, 11, 17) were significantly deviated from the Hardy–Weinberg Equilibrium (HWE) in more than three populations (in 6 pops for loci 3, 4 pops for loci 6 and 17 and 3 pops for locus 11). Given the assumptions of coalescent based analyses such as STRUCTURE and MIGRATE-N for HWE, we deleted the four markers that violated the HWE assumptions from all downstream analyses. 

**Figure 2:**
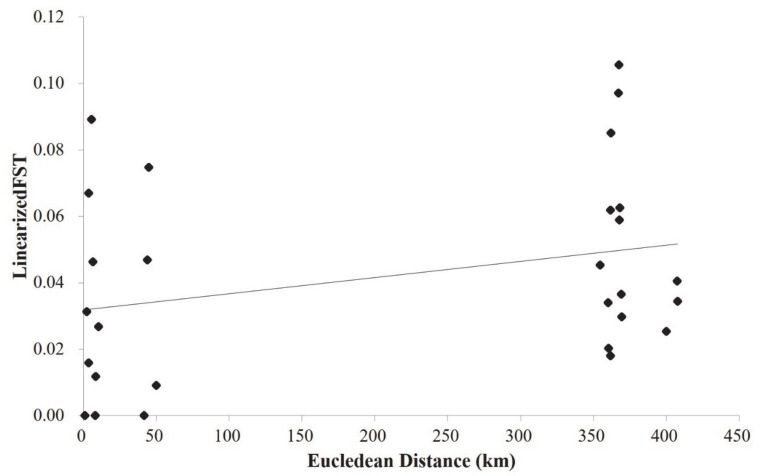
Result of the Mantel Test. The correlation between the logarithm of Euclidean distance (km) and Slatkin’s linearized F_ST_ (F_ST_/(1 − F_ST_)) for the population pairs among eleven populations of the three *Malus* taxa was marginally significant (r = 0.3, *p* = 0.06).

Overall, the diversity parameters (He and Na) averaged over 13 SSR loci varied among the 11 populations (Table 1). The Ala2 population harbored the least genetic diversity as revealed by both He (0.68, Ala2 and 0.78, AlaDom and AlmaDom) and Na (3.99, Ala2 and 4.42, Ala3; Table 1). Pairwise F_ST_ among 11 populations (56 population pairs) differed among population pairs, however, on average, the values were low (Table 2). On average, F_ST_ values were higher between population pairs from the two different regions (F_ST_ between east populations and west populations >~0.05; Table 2). Likewise, most wild apple populations were more genetically differentiated from the domesticated apple populations (Table 2). The two domesticated apple populations did not show population-level divergence from one another (Table 2). A Mantel test showed a positive correlation between the genetic differentiations (F_ST_) and the linearized geographic distances with a marginally significant statistical support (Euclidean distance; r = 0.3, *p* = 0.06; Figure 2).

We found the best K, i.e., the number of randomly mating subgroups, at K = 2 based on delta K values (Figure S1). However, K = 5 also showed a clustering pattern that cannot be ignored as the delta K value for K = 5 is comparably high compared to K = 2 (Figure S1; K2 and K5 referred to as K = 2 and K = 5, respectively, hereafter). Thus, we summarized and presented the results for both K2 and K5 (Figure 1). We also provided bar plots for K = 2 to 7 to show the varying cluster patterns as the K numbers grow (Figure S2). Overall, K2 and K5 showed a rather high level of admixture pattern between the clusters (Figure 1). K2 largely divided the 11 populations into the following two demes: (1) a deme mainly represented by blue ancestry (AlaDom, AlmaDom, M_nie, TalE and TalW) and (2) the other deme primarily represented by orange ancestry (the remaining six wild apple populations; Figure 1a). The admixture pattern was common in all 102 genotypes included in the analysis (Figure 1 and Figure S2). For K2, the number of genotypes with membership coefficients of 0.9 or higher was just 68 while remaining 34 genotypes showed shared ancestry. K5 showed the regional divergence between the east and the west, which is somewhat consistent with F_ST_ results (Figure 1b; Table 2). In the east region, Ala1 and Ala2 shared the similar ancestry, whereas Ala3 were genetically affiliated with both Ala1 and Ala2 and AlaDom (Figure 1b). Based on K5, the west region exhibited a more complex clustering pattern than the east. The three wild apple populations (Tal1, Tal2 and kokW) shared the ancestry, yet notably TalE and TalW showed a different ancestry pattern from the three (Figure 1b). K5 results revealed a more or less identical cluster pattern between the two domesticated apple populations in the east and the west with a slight introgression from TalE and TalW in the west (Figure 1b). M_nie genotypes were assigned primarily to the blue cluster as the cultivated apple genotypes were at K2, whereas the assignment pattern at K5 for M_nie was associated with the patterns revealed by both the cultivated apples and TalE and TalW (Figure 1a,b). The overall split pattern into four demes observed at K5 remained across varying K numbers except for K2 and K3 (Figure S2). 

**Figure 3:**
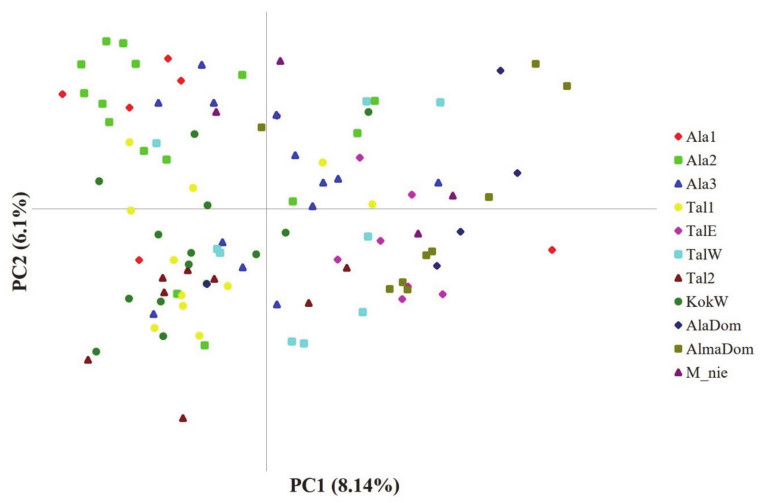
Principal Coordinate Analysis (PCoA) plot for 102 individuals from eleven populations of the three *Malus* taxa. The first two variance components are plotted. See Table 1 for population abbreviations, sample locations and sample sizes.

Contrary to the STRUCTURE results, PCoA results failed to show the clear cluster pattern of the 11 populations (Figure 3). The first two PCs only explained less than 15% of the total genetic variance harbored in our data (PC1 = 8.14%; PC2 = 6.1%). Accordingly, we plotted two more PCs with comparable amount of variance (PC3 and PC4) against each other and with the first two PCs, yet the assignment patterns were not easy to perceive from any of the plots. One of a few patterns prominently observed was that PC1 largely split the domesticated apples by positioning them at the right side (Figure 2). Although a couple of genotypes from TalW did not fit in, most TalE and TalW genotypes were grouped in the right side on the first PC axis (Figure 2). The molecular variance in our data was partitioned hierarchically with the two regional groups. The AMOVA result revealed that the genetic variance was primarily partitioned to the intra-population level (within individuals 75.9% and among individuals 18.3%; Table 3). Although the overall genetic divergence was very limited among populations and/or between groups, the populations were genetically more diverged within groups (F_SC_ = 0.035, *p* < 0.05) than across groups (F_CT_ = 0.024, *p* < 0.05; Table 3).

**Figure 4:**
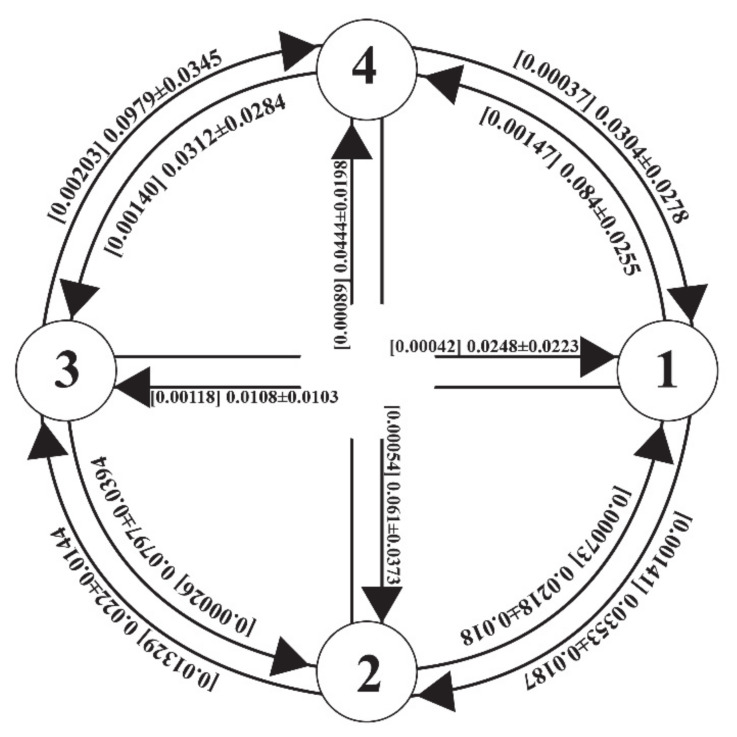
Pairwise contemporary and historical gene flow estimated as m, the number of immigrants per generation, among the four redefined groups of the three *Malus* taxa. The values in the square brackets are the historical gene flow. Groups 1, 2, 3 and 4 refer to Ala, TalKok, TalEW, Dom, respectively.

On average, the contemporary migration rates among the four re-arranged groups were much greater than the historical migration rates, although the differences varied greatly across the group pairs (Figure 4). Both the historical and contemporary migration rates turned out to be highly skewed for one direction and significantly different from zero based on the 95% credibility i.e., no overlap with zero for all migration rates estimated except for one historical migration rate, m3→2 (Table S1). The historical migration rates ranged from 0.0003 (m3→2) to 0.0133 (m2→3), suggesting an extreme asymmetry in historical migration rate, whereas the contemporary migration rates were less variable ranging from 0.011 to 0.098 (Figure 4). It is noticeable that group 1 (Ala) consisting of three east populations had a much greater number of emigrants than immigrants from the other three groups (Figure 4). The effective population size estimated as θ (4Neμ) was the largest at Ala (θ_1_ = 1.99) and the smallest at TalEW (θ_1_ = 0.85). The θ values observed in the MIGRATE-N result are comparable to the ones observed in long-lived shrubs (*Rhododendron* [57]; *Calothamnus* [58]; *Sibiraea* [59]). 

The G-W indices (M-ratio) for all ten populations (M_nie was excluded from the bottleneck analyses) were below the critical threshold, M = 0.73 estimated from the data using Critical_M (0.19 ≤ M-ratio ≤ 0.27; Table 4). The greatly reduced M-ratio values suggest historical population bottlenecks. In contrast, the recent population bottlenecks within the last few generations were not evident in most of the populations except for KokW (Table 4). Both Sign and Wilcoxon tests produced rather high *p* values for the nine populations failing to reject the null hypothesis i.e., the equilibrium state of population demography (Table 4). However, there was no significant mode shift observed in all eight wild apple populations including KokW, whereas the two domesticated apple populations exhibited a significant allele frequency mode shift compared to the equilibrium state (Table 4).

## 4. Discussion

Our molecular analyses of 13 SSRs revealed high level of admixture pattern across nearly all wild apple genotypes. Crops likely have a significant impact on the evolution of their wild relatives through inter- and intra-specific gene flow [7,60]. As shown in former studies, gene flow between crops and wild progenitors or the allied taxa in natural habitats is a widespread phenomenon [8,61,62,63]. *M. sieversii*, restrictedly distributed along the Tien Shan mountains, is the wild progenitor of cultivated apples [15,20]. Although the overall genetic diversity patterns were consistent with the previous observations [8,14,24,29], the results proposed a notable finding. The migration rates within the past few generations between cultivated apples and the wild progenitors were much greater than the long-term historical migration rates. Given the rapid growth of human activities within the recent past, the result suggests that geneflow among the cultivated and wild apples might have been strongly influenced by anthropogenic interventions such as cultivations near the wild habitats. 

Of seventeen loci, four (loci 3, 6, 11, 17) exhibited heterozygote deficiency (the observed heterozygosity of the four loci, Ho < 0.4; Ho for the remaining 13 loci >0.6) and were not in HWE. The reduced heterozygosity was likely derived from presence of null alleles. To avoid violating assumptions in coalescent-based approaches and biases in estimating F_ST_, we purged the four loci from all downstream analyses. Because we selectively took SSR loci from different chromosomes to ensure independence among loci, the linkage among the loci was not considered. Consistent with the previous observations, both wild and cultivated apples harbored a large amount of within-population genetic diversity as indicated by high He (Table 1). It is well recognized in plants that outcrossing species are genetically much more diverse than other mating systems i.e., selfing [64]. A vast majority angiosperm trees (over 95%) are indeed showing either obligatory outcrossing or mixed mating systems [65]. Thus, it is not surprising that the wild and cultivated apples are genetically highly diverse. The increased within-population genetic variation was likely driven by inter- and infra-specific hybridization among the closely related species with rather unclear boundaries [24]. The inflated genetic variation was further supported by the AMOVA results as most of the molecular variation was partitioned to below the individual levels (Table 3).

The lowered genetic variation among populations is pronounced in outcrossing trees [64,65]. Likewise, in our study, the level of genetic differentiation observed in wild and cultivated apple populations was low (Table 2). Besides the outcrossing nature of the apple trees, the high dispersal capability inferred from previous genetic studies [8,14] is likely one of the causal mechanisms for the lowered genetic divergence. Dispersers of apples trees are bees, birds and frugivorous mammals [8,20]. Frugivorous animal dispersers particularly large birds and/or mammals can transfer the seeds for long distances contributing to gene flow with increased dispersal capabilities among local populations and closely related taxa. Although the level of dispersal capacity in *Malus* taxa is not well investigated, it can roughly be inferred from an estimate (~1.5 km) of an allied species (*Prunus mahaleb*) in Rosaceae [66]. However, the extent of population divergence observed in our study was beyond the scope of frugivorous animal dispersal unless the dispersers are human. Thus, the surprisingly low level of population differentiations found in our study might primarily be driven by human-mediated transfer probably over a few hundred kilometers. Apart from the lowered average population divergences, we found that wild apple populations were much more differentiated from the domesticated apples than among the wild populations (Table 2). Indeed, the population divergence among the domesticated apple populations was nearly zero, suggesting that the local orchards likely grow the apples from a small number of cultivating stocks. 

The Bayesian- and distance-based assignment patterns (PCoA and STRUCTURE) were consistent with the population divergence pattern separating domesticated and wild apples (Figure 1 and Figure 3). As expected, the two domesticated apple populations exhibited nearly identical assignment patterns despite the long geographic distance, suggesting those populations have likely been cultivated from the same apple variety. The assignment pattern of the domesticated population in the west showed a more complex admixture pattern sharing the same ancestry with TalE and TalW (Figure 1). At K5, TalE and TalW populations presented a rather unique assignment pattern by being predominantly occupied by the pink genotype, which notably was observed in two eastern populations (Ala2 and Ala3; Figure 1). Such patterns may primarily be attributable to the high dispersal capacity explained also by the aforementioned population divergence pattern. 

The high level of dispersal potential might also be the causal mechanism of hybridization between the wild and cultivated apples. Crop-to-wild gene flow is relatively common in trees as they are generally equipped for long distance dispersal [67]. Recent studies explored the introgression and hybridization between the crops and the wild relatives and revealed that about 20% of the studied crops showed certain level of crop-to-wild gene flow [63,67,68,69]. It is well appreciated that the apple trees are obligatory outcrossing plants with frequent intra- and inter-specific geneflow [8,20]. It is not surprising that all wild apple populations in our study exhibited some extent of admixture with the cultivated apples (Figure 1). Given that K2 largely consists of the two subgroups represented by wild and cultivated apple groups, the admixture ancestries between crop and wild apples can be inferred from the K2 assignment pattern. Based on the membership coefficient at K2, the rates of admixture vary from 0.1 (TalE) to 0.4 (Ala3), indicating an evident admixture between the wild and cultivated apples in the Tien Shan area. 

Unexpectedly, the endangered wild apple, *M. niedzwetzkyana* (red fleshed apple), was assigned primarily to the cluster of the cultivated apple genotypes, although the assignment pattern was more complex at K5 sharing its genetic affinity the highest with TalE and TalW (Figure 1a,b). The red fleshed apple is regarded as an important genetic resources of apple breeding programs due to its distinctive pink coloration with the high anthocyanin content [70], yet the plant only resides in a few extremely restricted areas making it vulnerable to extinction [71]. The species is currently listed as “Endangered” on the IUCN RED LIST (https://www.iucnredlist.org). Although the statistical power is low due to small sample size for the species, our study demonstrated that the genetic integrity of *M. niedzwetzkyana* might have been eroded by cultivated apples, at least in Kazakhstan. Thus, there is an urgent need to monitor the level of admixture between the crop apples and *M. niedzwetzkyana* throughout its distribution. 

It surely is worthwhile to examine the genetic structure and the population divergences as a proxy of gene flow, yet the genetic structure per se is not the direct measure of gene flow [72]. Given the challenges to directly measuring the gene flow in natural populations, an indirect measure of gene flow has been developed and widely used in the various eco- and evolutionary studies [72,73]. To further infer gene flow between the crop apples and the wild progenitor apples, we estimated both the historical and contemporary migration rates among the four rearranged groups. Consistent with the assignment results, there was clear evidence of gene flow between the cultivated apples and the wild apples (Figure 4; Table S1). Indeed, the migration rates between the cultivated (Dom) and wild apples were comparable to the ones among the wild apple groups (Figure 4; Table S1). Furthermore, the contemporary gene flow was much greater than the historical gene flow, proposing a likely influence of the escalated anthropogenic transfer within the recent past, although the differences varied greatly across the group pairs (Figure 4). The increased gene flow between the wild and crop apples surely raises the risk of genetic swamping that could lead to a local extinction. Harmful effects of hybridization are well appreciated by conservation biologists [3], as anthropogenic activities promote the hybridization and consequently increase extinction risks [5]. 

Coupled with the risk of genetic swamping, there is also concern for demographic effects such as population bottlenecks. Based on the G-W indices, most populations might have experienced a series of population declines in the past, yet no recent bottlenecks were observed for the wild apple populations (Table 4). It alleviates apprehensions for extinction risks that might be facilitated by the bottleneck effects. Our study suggested that the crop-to-wild gene flow is predominant in wild apples along the Tian Shan, Kazakhstan. The hybridization is likely leading to gradual increase in the extinction risk for the wild progenitor of apples. Extinction of the wild apple itself may negatively influence biodiversity of natural habitats. In addition, the wild relatives of the cultivated apple are invaluable resources for improving the crops’ performances i.e., pest resistance and production. Therefore, conserving the wild relatives of major crops is of great importance to maintain diversity pools as means of breeding programs.

## Figures and Tables

**Table 1:** Locations, sample sizes and summary statistics of genetic diversity for eleven populations of the three closely related *Malus* taxa (*M. sieversii*, *M. domestica* and *M. niedzwetzkyana*). Group abbreviation—an abbreviation given to a cluster to which the population is assigned for the migration rate estimates. N—Sample size. Lat and Lon—geographic coordinates. He—mean expected heterozygosity over 13 nuclear microsatellite markers (nrSSRs). Na—mean number of alleles over 13 nrSSRs with rarefaction. sd—standard deviation. Se—standard error.

Species	Region	Location	Abbreviation	Group Abbreviation	N	Lon	Lat	He (±sd)	Na (±se)
*sieversii*	East	Lepsi Rever side, Almaty, KAZ	Ala1	Ala	7	45.5--	80.6--	0.73 (0.14)	4.12 (0.28)
	East	Mt. Lepsy, Almaty, KAZ	Ala2	Ala	15	45.5--	80.7--	0.68 (0.23)	3.99 (0.31)
	East	South-western Lepsy, Almaty, KAZ	Ala3	Ala	13	45.5--	80.5--	0.77 (0.16)	4.42 (0.28)
	West	Mt. Lepsy (southern), KAZ	Tal1	Tal1	11	45.5--	80.5--	0.75 (0.18)	4.26 (0.28)
	West	Mt. Ryskulov (southern), Almaty, KAZ	TalE	TalEW	7	43.2--	77.2--	0.75 (0.16)	4.24 (0.29)
	West	South-western Orman (east Valley), Almaty, KAZ	TalW	TalEW	9	43.2--	77.3--	0.74 (0.24)	4.34 (0.34)
	West	South-western Orman (West valley), Almaty, KAZ	Tal2	Tal2	8	43.2--	77.3--	0.71 (0.22)	4.27 (0.34)
	West	Mt. Ryskulova (eastern), Almaty, KAZ	KokW	KokW	14	43.2--	77.3--	0.76 (0.19)	4.39 (0.30)
*domestica*	-	Koklaisay, Almaty, KAZ	AlaDom	Dom	5	43.1--	76.8--	0.78 (0.12)	4.37 (0.27)
		Belbulak, Almaty, KAZ	AlmaDom	Dom	9	43.3--	77.0--	0.78 (0.09)	4.33 (0.23)
*niedzwetzkyana*	-	Southern Bayzeren, Almaty, KAZ	M_nie	-	4	45.6--	80.6--	0.77 (0.19)	4.31 (0.38)

**Table 2:** Estimated pairwise F_ST_ values from 13 nrSSRs among eleven populations of the three *Malus* taxa. See Table 1 for abbreviations of population locations and sample sizes. All values were significantly different from 0 at the *p* < 0.05 level except for the values with ns (*p* > 0.05).

	Ala1	Ala2	Ala3	Tal1	TalE	TalW	Tal2	KokW	AlaDom	AlmaDom	M_nie
Ala1	0.00										
Ala2	0.03 ns	0.00									
Ala3	0.01 ns	0.04	0.00								
Tal1	0.05	0.04	0.03	0.00							
TalE	0.10	0.11	0.05	0.07	0.00						
TalW	0.07	0.07	0.03	0.04	0.001 ns	0.00					
Tal2	0.07	0.09	0.05	0.02 ns	0.09	0.05	0.00				
KokW	0.05	0.04	0.03	0.01	0.08	0.05	0.02	0.00			
AlaDom	**0.08**	**0.11**	**0.04 ns**	**0.07**	**0.051 ns**	**0.07**	**0.10**	**0.09**	0.00		
AlmaDom	**0.10**	**0.12**	**0.07**	**0.09**	**0.05**	**0.09**	**0.12**	**0.09**	0.02 ns	0.00	
M_nie	0.08	0.07	0.05 ns	0.07	0.03 ns	0.05 ns	0.13	0.07	0.06 ns	0.07	0.00

The values in bold indicate the pairwise F_ST_ values between the wild apple populations and the domesticated apple populations.

**Table 3:** Summary on the analysis of molecular variance (AMOVA) in the three *Malus* species across 13 nuclear SSRs. For the hierarchical partitioning of genetic variance, groups were defined based on the species, geography and the genetic clusters identified from the STRUCTURE. All variance components were statistically significant (*p* < 0.01).

Source	Sum of Squares	Variance Components	Percentage of Variation	Fixation Index
Among groups (FCT)	46.41	0.18	3.50	0.035
Among populations within groups (FSC)	56.74	0.12	2.29	0.024
Among individuals within populations (FIS)	527.85	0.95	18.31	0.194
Within individuals (FIT)	399.50	3.93	75.91	0.240

**Table 4:** Results summary of recent and past bottlenecks in the eleven populations of the three *Malus* taxa: the G-W index is the Garza–Williamson index, known as the M-ratio. The significant *p*-values of Sign and Wilcoxon tests are estimated from the tests for excess or deficit of heterozygosity across nine microsatellite loci under the stepwise mutation model (IAM) and (SMM) mutation models.

Population	G-W Index (±sd)	P (Sign Test)		P (Wilcoxon Test)		Mode Shift
		IAM	SMM	IAM	SMM	
Ala1	0.22 (0.08)	0.51	0.19	1.00	0.15	no
Ala2	0.27 (0.10)	0.10	**0.01**	0.74	**0.01**	no
Ala3	0.25 (0.10)	0.15	0.48	0.05	0.54	no
Tal1	0.23 (0.08)	**0.05**	0.43	0.09	1.00	no
TalE	0.19 (0.08)	0.14	0.55	0.09	0.64	no
TalW	0.23 (0.11)	0.31	0.12	0.24	0.24	no
Tal2	0.21 (0.08)	0.25	**0.01**	0.38	0.02	no
KokW	0.23 (0.08)	**0.01**	**0.04**	**0.02**	0.15	no
AlaDom	0.20 (0.08)	0.19	0.54	**0.04**	0.84	yes
AlmaDom	0.27 (0.11)	0.06	0.26	**0.00**	0.95	yes

Values in bold are statistically significant by *p* < 0.05.

## Data Availability

Not applicable.

## Supplementary Materials

The following are available online at https://www.mdpi.com/2073-4425/12/1/104/s1, Table S1: summary of pairwise contemporary and historical gene flow among the four redefined groups of the three *Malus* taxa. m (migration rate) is the number of immigrants per generation. H stands for historical while C refers to contemporary. Mean HNm refers to the historical effective migration rate (Nem) that is the migration rate given the effective population size. Cm/Hm ratio is also presented as a measure of difference between the historical and contemporary gene flow. See Table 1 for the population acronyms. Table S2: Detailed information on 17 microsatellite loci utilized in the study. Table S3: Summary of PCR amplification conditions. Figure S1: Delta K plot. Delta K values for each of K clusters from 2 to 9 were estimated by method of Evanno et al. (2005). Figure S2: Bar plots of K = 2 to K = 7 from the Bayesian model-based group assignment on the 11 populations of the three *Malus* taxa with 13 nuclear SSRs. Populations are separated by solid vertical black lines. Colors represent assignments of loci into each of the 2–7 estimated groups. See Table 1 for population abbreviations.
