# Supplementary material for: Genetic Admixture in the Population of Wild Apple (Malus sieversii) from the Tien Shan Mountains, Kazakhstan
Source: Genes (Basel). 2021 Jan 15;12(1):104. doi: 10.3390/genes12010104 (PMC7829876; doi:10.3390/genes12010104)
Supplement: Supplementary file 1 [file genes-12-00104-s001.pdf]

Supplementary Information

Increased risk of genetic swamping in the progenitor of the domesticated apple (*Malus sieversii*) from the diversity hotspot, Tien Shan, Kazakhstan

Table 1. Summary of pairwise contemporary and historical gene flow among the four redefined groups of the three *Malus* taxa. m (migration rate) is the number of immigrants per generation. H stands for historical while C refers to contemporary. mean HNm refers to the historical effective migration rate (Nem) that is the migration rate given the effective population size. Cm/Hm ratio was also presented as a measure of difference between the historical and contemporary gene flow. See Table 1 for the population acronyms.

	**mean HNm**	**mean Hm**	**lower Hm**	**upper Hm**	**mean Cm**	**95% CI of Cm**	**Cm/Hm**
m_TalKok->Ala	1.795163	0.000726	0.000503	0.000925	0.021800	0.018000	30.03
m_TalEW->Ala	1.029359	0.000416	0.000161	0.000644	0.024800	0.022300	59.58
m_Dom->Ala	0.910013	0.000368	0.000141	0.000563	0.030400	0.027800	82.61
m_Ala->TalKol	1.898579	0.001408	0.001116	0.001661	0.035300	0.018700	25.07
m_TalEW->TalKok	0.356213	0.000264	0.000000	0.000490	0.079700	0.039400	301.73
m_Dom->TalKok	0.723443	0.000536	0.000272	0.000790	0.061000	0.037300	113.71
m _Ala->TalEW	0.540516	0.001184	0.000702	0.001638	0.010800	0.010300	9.12
m _TalKol->TalEW	4.520675	0.013293	0.009904	0.015166	0.022000	0.014400	1.65
m _Dom->TalEW	0.632382	0.001385	0.000843	0.001919	0.031200	0.028400	22.52
m _Ala->Dom	0.928644	0.001466	0.000715	0.001669	0.084000	0.025500	57.29
m _TalKok->Dom	0.566246	0.000894	0.000477	0.001272	0.044400	0.019800	49.66
m _TalEW->Dom	1.283492	0.002027	0.001033	0.002821	0.097900	0.034500	48.31

Table S2. Detailed information on 17 microsatellite loci utilized in the study

No.	SSR primer	Fluorescently dye	Sequence	Allele range	Motif	Reference
1	MdBHLH3	FAM	CAACTCCCCTTATTCTTCTTCTCTC<br>CACCTGACCTTCTCTCTACCTCTAC	-	AG	Faramarzi et al., [31]
2	NZmsPal8	HEX	GGCACAAGCACAAGGAAACA<br>GTTTGAGCCAGTCCATTTTTCCCTAT	-	AG	
3	NzmsPal13	FAM	TCGATGAACAAGGCCCAAAG<br>GTTTAGGGGGACGGAAGGAACAAG	-	AG	
4	NzmsPal36	HEX	CCTCAACAAATATAAGACTCTCTC<br>GTTTCTCCACTCTGTCCGTACATT	-	AG	
5	P188-CH03d07	FAM	CAAATCAATGCAAAACTGTCA<br>GGCTTCTGGCCATGATTTTA	186-226	AG	Dong, Y. et al., [32]
6	P290-Hi03a10	HEX	GGACCTGCTTCCCCTTATTC<br>GTTTCAGGGAACTTGTTTGATGG	206-290	AG	
7	P300-Hi08h12	FAM	GAAGGAAATCATCATCAAGACG<br>GTTTCAAGACCATGGAACAACTTGG	151-203	GAA	
8	CH05g08	FAM	CCAAGACCAAGGCAACATTT<br>CCCTTCACCTCATTCTCACC	161–179	AG	Silfverberg-Dilworth, Eve, et al., [33]
9	CH03e03	HEX	GCACATTCTGCCTTATCTTGG<br>AAAACCCACAAATAGCGCC	106–216	AG	
10	Hi23g02	FAM	TTTTCCAGGATATACTACCCTTCC<br>GTTTCTTCGAGGTCAGGGTTTG	230–257	CAA	
11	CH03a09	HEX	GCCAGGTGTGACTCCTTCTC	125–143	AG	

			CTGCAGCTGCTGAAACTGG		
12	CH05a05	FAM	TGTATCAGTGGTTTGCATGAAC<br>GCAACTCCCAACTCTTCTTTCT	198–230	AG
13	CH01c06	HEX	TTCCCCATCATCGATCTCTC<br>AAACTGAAGCCATGAGGGC	146–188	AG
14	CH01f03b	FAM	GAGAAGCAAATGCAAAACCC<br>CTCCCCGGCTCCTATTCTAC	137-183	AG
15	CH03c02	HEX	TCACTATTTACGGGATCAAGCA<br>GTGCAGAGTCTTTGACAAGGC	166-136	AG
16	CH04c07	FAM	GGCCTTCCATGTCTCAGAAG<br>CCTCATGCCCTCCACTAACA	98-135	AG
17	CH02a03	HEX	AGAAGTTTTCACGGGTGCC<br>TGGAGACATGCAGAATGGAG	144-170	AG

Table S3. Summary of PCR amplification conditions

Primer number	Reference	PCR condition
1,2,3,4	Faramarzi et al., [31]	94 °C 2 min 30 sec, 4 cycles: 94 °C 30 sec, 65 °C 1 min, 72 °C 1 min, 30 cycle: 94 °C 30 sec, 60 °C 1 min and 72 °C 1 min; 72 °C 5 min (dropping 1 °C per cycle)
5,6,7	Dong, Y. et al., [32]	94 °C 4 min, 4 cycles: 94 °C 50 sec, 65 °C 55 sec, 72 °C 1 min, 30 cycle: 94 °C 50 sec, 65 °C 55 sec and 72 °C 1 min; 72 °C 5 min (dropping 1 °C per cycle)
8,9,10,12	Silfverberg-Dilworth, Eve, et al., [33]	94 °C 5 min, 10 cycles: 94 °C 30 sec, 58–53 °C 45 sec (dropping 0.5 °C per cycle) and 72 °C 1 min, 25 cycles: 94 °C 30 sec, 53 °C 45 sec and 72 °C 1 min; 72 °C 15 sec
11,13,14,15,16,17		94 °C 5 min, 38 cycles: 94 °C 30 sec, 60 °C 30 sec and 72 °C 1 min, 72 °C 10 min

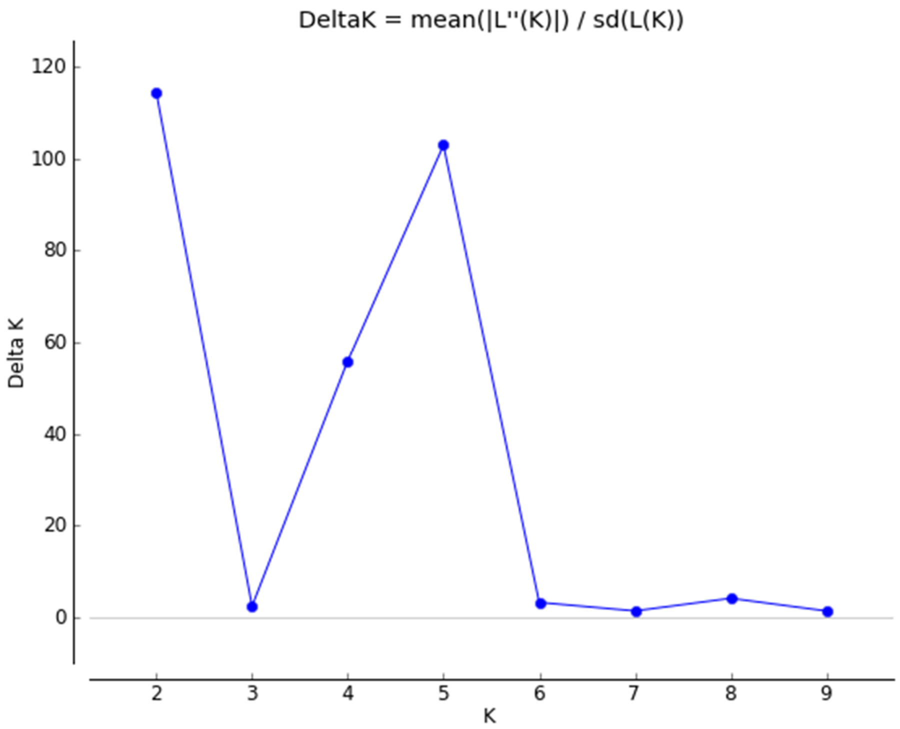

Figure S1. Delta K plot. Delta K values for each of K clusters from 2 to 9 were estimated by method of Evanno et al.,[43].

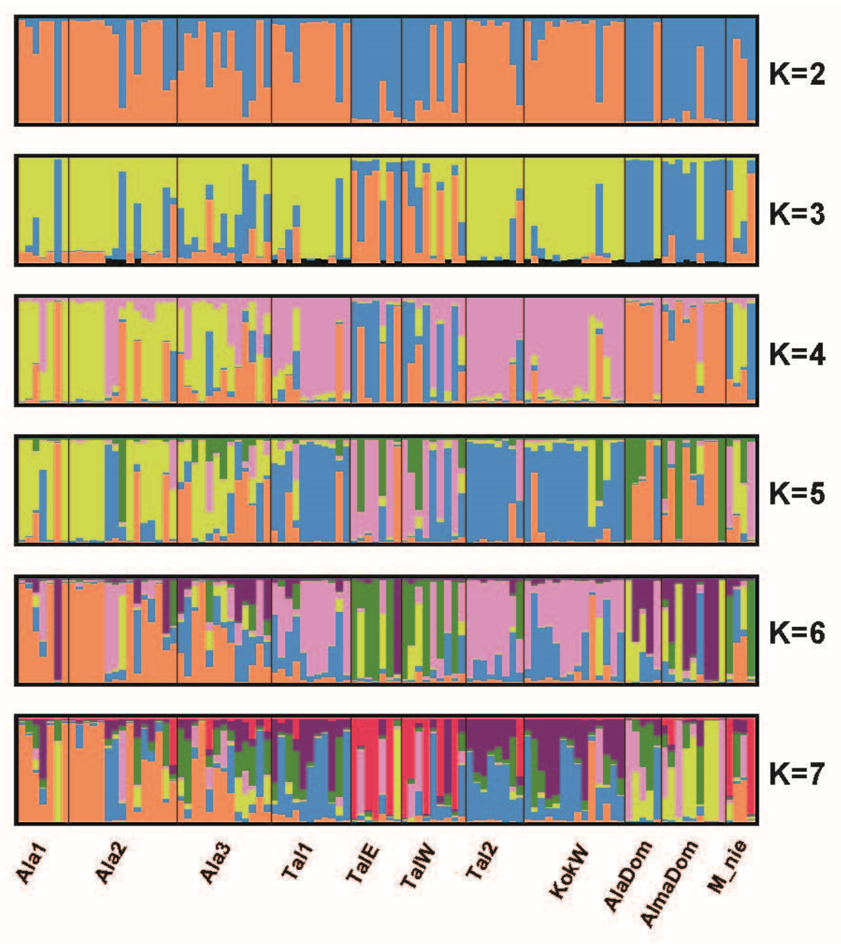

Figure S2. Bar plots of K = 2 to K = 7 from the Bayesian model-based group assignment on the 11 populations of the three *Malus* taxa with 13 nuclear SSRs. Populations are separated by solid vertical black lines. Colors represent assignments of loci into each of the 2–7 estimated groups. See Table 1 for population abbreviations.
